# Significance of PIK3CA Mutations in Patients with Early Breast Cancer Treated with Adjuvant Chemotherapy: A Hellenic Cooperative Oncology Group (HeCOG) Study

**DOI:** 10.1371/journal.pone.0140293

**Published:** 2015-10-09

**Authors:** George Papaxoinis, Vassiliki Kotoula, Zoi Alexopoulou, Konstantine T. Kalogeras, Flora Zagouri, Eleni Timotheadou, Helen Gogas, George Pentheroudakis, Christos Christodoulou, Angelos Koutras, Dimitrios Bafaloukos, Gerasimos Aravantinos, Pavlos Papakostas, Elpida Charalambous, Kyriaki Papadopoulou, Ioannis Varthalitis, Ioannis Efstratiou, Thomas Zaramboukas, Helen Patsea, Chrisoula D. Scopa, Maria Skondra, Paris Kosmidis, Dimitrios Pectasides, George Fountzilas

**Affiliations:** 1 Oncology Section, Second Department of Internal Medicine, “Hippokration” Hospital, Athens, Greece; 2 Department of Pathology, Aristotle University of Thessaloniki, Faculty of Medicine, Thessaloniki, Greece; 3 Laboratory of Molecular Oncology, Hellenic Foundation for Cancer Research, Aristotle University of Thessaloniki, Faculty of Medicine, Thessaloniki, Greece; 4 Health Data Specialists Ltd, Dept of Biostatistics, Athens, Greece; 5 Translational Research Section, Hellenic Cooperative Oncology Group, Data Office, Athens, Greece; 6 Department of Clinical Therapeutics, “Alexandra” Hospital, University of Athens School of Medicine, Athens, Greece; 7 Department of Medical Oncology, “Papageorgiou” Hospital, Aristotle University of Thessaloniki, Faculty of Medicine, Thessaloniki, Greece; 8 First Department of Medicine, “Laiko” General Hospital, University of Athens School of Medicine, Athens, Greece; 9 Department of Medical Oncology, Ioannina University Hospital, Ioannina, Greece; 10 Second Department of Medical Oncology, “Metropolitan” Hospital, Piraeus, Greece; 11 Division of Oncology, Department of Medicine, University Hospital, University of Patras Medical School, Patras, Greece; 12 First Department of Medical Oncology, “Metropolitan” Hospital, Piraeus, Greece; 13 Second Department of Medical Oncology, “Agii Anargiri” Cancer Hospital, Athens, Greece; 14 Oncology Unit, “Hippokration” Hospital, Athens, Greece; 15 Oncology Department, General Hospital of Chania, Crete, Greece; 16 Department of Pathology, "Papageorgiou" Hospital, Thessaloniki, Greece; 17 Department of Pathology, IASSO General Hospital, Athens, Greece; 18 Department of Pathology, University Hospital, University of Patras Medical School, Patras, Greece; 19 Second Department of Medical Oncology, Hygeia Hospital, Athens, Greece; Florida International University, UNITED STATES

## Abstract

**Background:**

The PI3K-AKT pathway is frequently activated in breast cancer. PIK3CA mutations are most frequently found in the helical (exon 9) and kinase (exon 20) domains of this protein. The aim of the present study was to examine the role of different types of PIK3CA mutations in combination with molecular biomarkers related to PI3K-AKT signaling in patients with early breast cancer.

**Methods:**

Tumor tissue samples from 1008 early breast cancer patients treated with adjuvant chemotherapy in two similar randomized trials of HeCOG were examined. Tumors were subtyped with immunohistochemistry (IHC) and FISH for ER, PgR, Ki67, HER2 and androgen receptor (AR). PIK3CA mutations were analyzed by Sanger sequencing (exon 20) and qPCR (exon 9) (Sanger/qPCR mutations). In 610 cases, next generation sequencing (NGS) PIK3CA mutation data were also available. PIK3CA mutations and PTEN protein expression (IHC) were analyzed in luminal tumors (ER and/or PgR positive), molecular apocrine carcinomas (MAC; ER/PgR negative / AR positive) and hormone receptor (ER/PgR/AR) negative tumors.

**Results:**

PIK3CA mutations were detected in 235/1008 tumors (23%) with Sanger/qPCR and in 149/610 tumors (24%) with NGS. Concordance between the two methods was good with a Kappa coefficient of 0.76 (95% CI 0.69–0.82). Lobular histology, low tumor grade and luminal A tumors were associated with helical domain mutations (PIK3CAhel), while luminal B with kinase domain mutations (PIK3CAkin). The overall incidence of PIK3CA mutations was higher in luminal as compared to MAC and hormone receptor negative tumors (p = 0.004). Disease-free and overall survival did not significantly differ with respect to PIK3CA mutation presence and type. However, a statistically significant interaction between PIK3CA mutation status and PTEN low protein expression with regard to prognosis was identified.

**Conclusions:**

The present study did not show any prognostic significance of specific PIK3CA mutations in a large group of predominantly lymph-node positive breast cancer women treated with adjuvant chemotherapy. Further analyses in larger cohorts are warranted to investigate possible differential effect of distinct PIK3CA mutations in small subgroups of patients.

## Introduction

Phosphoinositide 3-kinase–protein kinase B (PI3K-AKT) is an important intracellular pathway regulating numerous cellular activities, mostly survival, proliferation, growth and glycogen metabolism. It is activated in many different tumor types and its therapeutic targeting has attracted significant interest **[1]**. PI3K-AKT signaling is an important driving pathway in breast cancer, triggered by growth factor receptor activation, such as insulin-like growth factor receptors (IGF-R), epidermal growth factor receptor (EGFR) and HER2, by steroid hormone receptors, estrogen (ER), progesterone receptors (PgR), as well as by genetic aberrations of some of its components, more frequently HER2 amplification, PIK3CA mutations and phosphatase and tensin homolog (PTEN) loss **[2]**. Also, evidence of cross-talk between androgen receptors (AR) and the PI3K-AKT pathway has been reported **[3–5].** Pathway activation leads to phosphorylation of AKT at two different amino acid residues, pAKT473 and pAKT308. AKT directly phosphorylates and activates mTOR. PTEN is a lipid phosphatase that inhibits AKT activation **[6]**.

Adding more complexity to the model, different PIK3CA mutations exist, with two common hotspot areas, the helical (exon 9, PIK3CAhel) and the kinase (exon 20, PIK3CAkin). The exact mechanism by which these different types of mutations affect the PI3K pathway is not yet clarified. Both PIK3CAhel and PIK3CAkin were found to exert gain-of-function and transforming activity. However, more recent data revealed that each type of mutation might lead to different interactions with protein partners and different tumorigenic potential in animal models **[7]**.

In the present study, different types of PIK3CA mutations were analyzed with regard to their frequency, their association with basic characteristics and their prognostic significance in patients with early breast cancer. Also, the possible interactions of these mutations with multiple other biomarkers, such as EGFR, AR and members of the IGF and PI3K-AKT pathway, described in our previous reports **[8–10]**, were investigated.

## Patients and Methods

### Patients

In this prospective/retrospective translational research study, patients from two randomized adjuvant chemotherapy trials were included. The characteristics of these trials are described in brief in **S1 Table** and detailed information about them can be found in the respective original papers **[11–13]**. At that time, trastuzumab was not licensed as adjuvant treatment; however it was administered in HER2 positive patients upon relapse **[14]**. The present translational research protocol was approved by the Bioethics Committee of the Aristotle University of Thessaloniki School of Medicine and patients provided written informed consent for the use of their biological material for future research purposes.

### Tumor tissue samples, processing, methods and biomarkers

For all methods, formalin-fixed paraffin-embedded (FFPE) tumor tissue samples were used. Tissue blocks were collected retrospectively in the first trial (HE10/97) and prospectively in the second (HE10/00). Tumors were histologically evaluated on hematoxcylin & eosin (H&E) sections for tumor presence and marked for the most tumor dense areas. Tumor cell content (TCC) was assessed as the ratio of cancer vs. non-cancer cells in these areas, which were manually macrodissected from 10 micron unstained sections for subsequent DNA extraction. Next, a second H&E evaluation round was employed for obtaining cores for tissue microarray (TMA) construction, which were used for immunohistochemistry (IHC) and fluorescent in situ hybridization (FISH). TMA blocks were constructed with a manual tissue microarrayer (Beecher Instruments, Sun Prairie, WI), using 2 cores per tumor, each 1.5 mm in diameter, along with orientation and IHC control sample cores. Cases with low tumor tissue availability were represented in TMAs only.

Tumors (n = 1008) were subtyped with IHC on 3 micron sections for ER, PgR, Ki67 and HER2; HER2 FISH was applied on 5 micron sections for determining HER2 status in ambiguous cases. Thus, tumors were classified as Luminal A (ER/PgR positive and Ki67 <14%); Luminal B (ER/PgR positive and Ki67 ≥14%); Luminal-HER2 (ER/PgR positive and HER2 positive); HER2-enriched (ER/PgR negative and HER2 positive); and, triple-negative (TNBC, ER/PgR/HER2 negative) **[15]**. In addition to classic subtyping, IHC for the androgen receptor (AR) was also performed for the classification of AR positive, ER/PgR negative carcinomas as molecular apocrine (MAC), as described by Lakis et al **[9]**. Finally, IHC biomarkers of the PI3K (EGFR, PTEN, pAKT473 [cytoplasmic and nuclear], pAKT308, mTOR) and insulin-like growth factor (IGF) pathways were examined, as described by Lazarides et al **[8]** and Mountzios et al **[10]**.

### PIK3CA mutations

DNA was extracted with dual nucleic acid extraction using silica-coated magnetic beads (Versant Tissue Preparation Reagents, Siemens Healthcare Diagnostics, Tarrytown, NY) from 1008 samples according to the manufacturer’s instructions.

**Fig 1:**
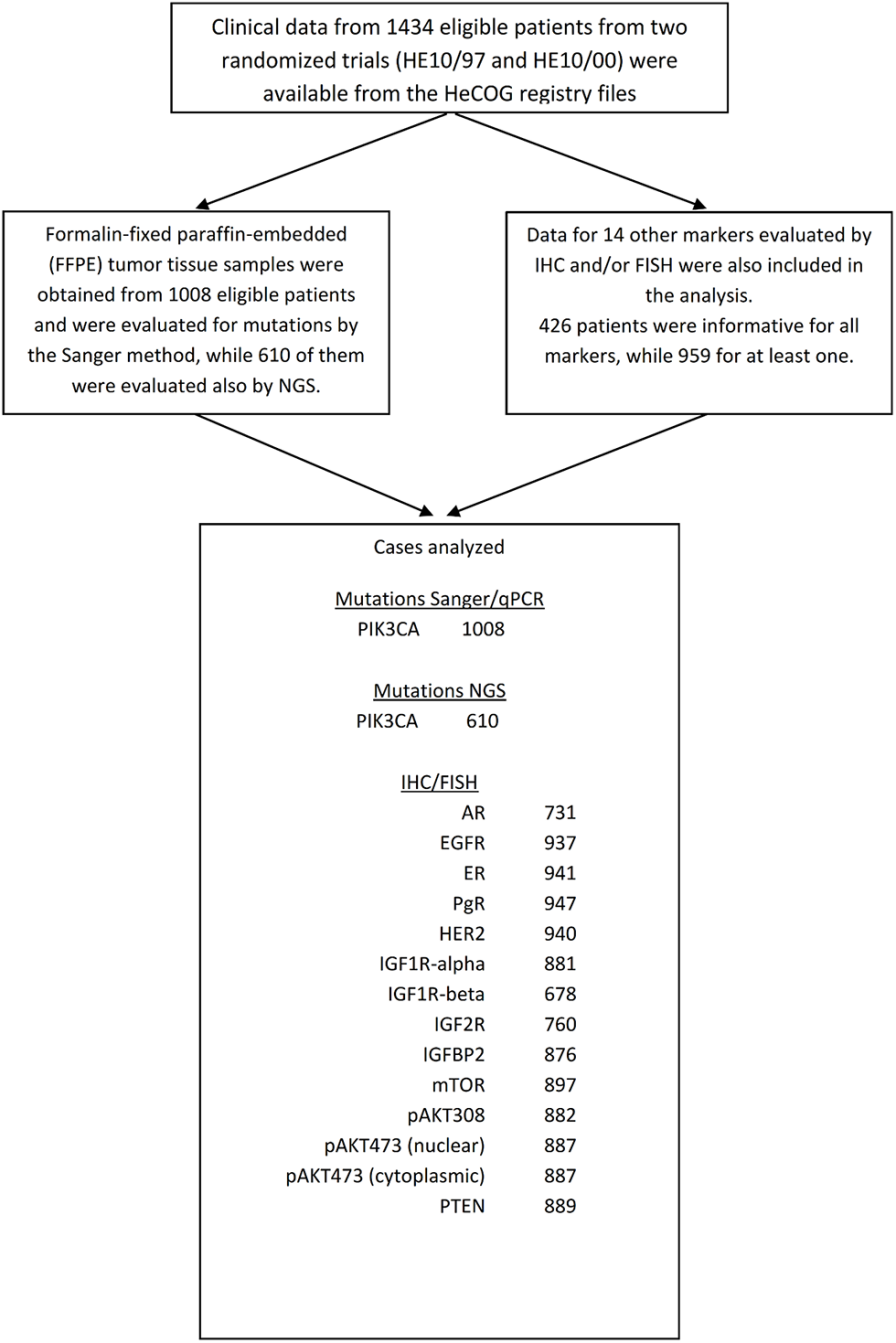
REMARK diagram.

PIK3CA mutations were investigated with classic methods and with next generation sequencing (NGS) (**Fig 1**). Classic methods included Taqman-MGB-SNP genotyping assays (Applied Biosystems, Foster City, CA) for E542K and E545K (exon 10, coding exon 9) and subsequent allelic discrimination in a 7900HT (Applied Biosystems) real time PCR (qPCR) system (Razis, Bobos et al. 2011). These methods were applied on 1008 tumor FFPE DNA samples. For mutations in exon 21 (coding exon 20), Sanger sequencing (Sanger) on nested PCR products with M-13 coupled primers was performed in 10 ul reactions with the Big Dye Teminator kit v.1.1 (Applied Biosystems). Sequences were visualized upon capillary electrophoresis in an ABI3130XL genetic analyzer, and were initially called with the Sequencing Analysis software v.5.2. The nested intron-spanning gene-specific primer pair was 5’-TTTTCTCAATGATGCTTGGCT-3’ (forward) and 5’-CCTGCTGAGAGTTATTAACAGT-3’ (reverse). This method switch was considered necessary for exon 20 in order to avoid detecting sequences from the highly homologous PIK3CA pseudogene on chromosome 22 (LOC100422375).

NGS was applied on libraries constructed with the Ampliseq Library Kit v.2.0 and Ion Xpress barcodes, according to the manufacturer’s instructions (Life Technologies / Ion Torrent, Carlsbad, CA), as previously described **[16]**. In total, 610 tumor DNA samples were examined with this method. PIK3CA coding regions were targeted with 9 amplicons covering the following genomic areas (positions according to GRCh37 / hg19: exon 2 (coding exon 1) (178916463…178916617 and 178916552…178916726); exon 10 (coding 9) (178935907…178936051 and 178935994…178936131); exon 11 (coding 10) (178936910…178937071); exon 20 (coding 19) (178948084…178948232); exon 21 (coding 20) (178951797…178951968, 178951918…178952092 and 178952020…178952194). Variants were initially called with Variant Caller and further analyzed with Ion Reporter v.6. Variants were excluded for Ion Reporter p value quality metric >0.0001; position coverage <125 and variant coverage <50; variant allele coverage vs. position coverage <6%; and, if they were non-annotated. Thus, variants at a frequency of 40% in low performing amplicons were accepted (worst case variants). Amino acid changing variants in coding regions (missense and nonsense) with minor allele frequency <1% if annotated single nucleotide polymorphisms (SNPs) were considered as mutations.

### Statistical analysis

Categorical data are displayed as frequencies and corresponding percentages. Possible associations between PIK3CA and clinical characteristics or other markers were investigated by performing Chi-square tests. The presence of PIK3CA mutations, as obtained with Sanger/qPCR and NGS, was compared through the calculation of a) the concordance rate along with the 95% confidence intervals for the estimation, defined as the proportion of the equal results, and b) the Cohen’s Kappa coefficient of agreement.

Disease-free survival (DFS) was measured from the date of diagnosis until verified disease progression, death or last contact, whichever occurred first, while overall survival (OS) from diagnosis until death from any cause or date of last contact. Time-to-event distributions were estimated using Kaplan-Meier curves, while log-rank tests and univariate Cox regression analyses were used for assessing statistically significant differences and reporting hazard ratios, respectively. Univariate Cox regression analyses with interactions were performed in order to investigate for possible differences of PIK3CA effects on DFS and OS among the levels of other variables. The identified interactions in the univariate setting between PIK3CA and other markers were adjusted for demographic, clinical and treatment characteristics. Multivariate Cox regression analysis was performed, with a backward selection procedure with a removal criterion of p >0.10 in order to identify significant factors. The clinical variables that were assessed in multivariate analysis were: adjuvant hormonotherapy (Yes vs. No), adjuvant radiotherapy (Yes vs. No), age (<50 vs. ≥50 years), paclitaxel treatment (Yes vs. No), menopausal status (post vs. pre), number of positive lymph nodes (0 vs. 1–3 vs. >4), tumor size (≤2cm vs. >2cm), surgery (BCS vs. MRM) and molecular subtype (Luminal A vs. Luminal B vs. Luminal-HER2 vs. HER2-enriched vs TNBC).

In univariate analysis, significance was determined at the level of 5% and all tests were two-sided. The statistical analysis complied with the reporting recommendations for tumor marker prognostic studies **[17]** and was performed using the SAS software (SAS for Windows, version 9.3, SAS Institute Inc., Cary, NC).

## Results

### PIK3CA mutation analysis

**Fig 2:**
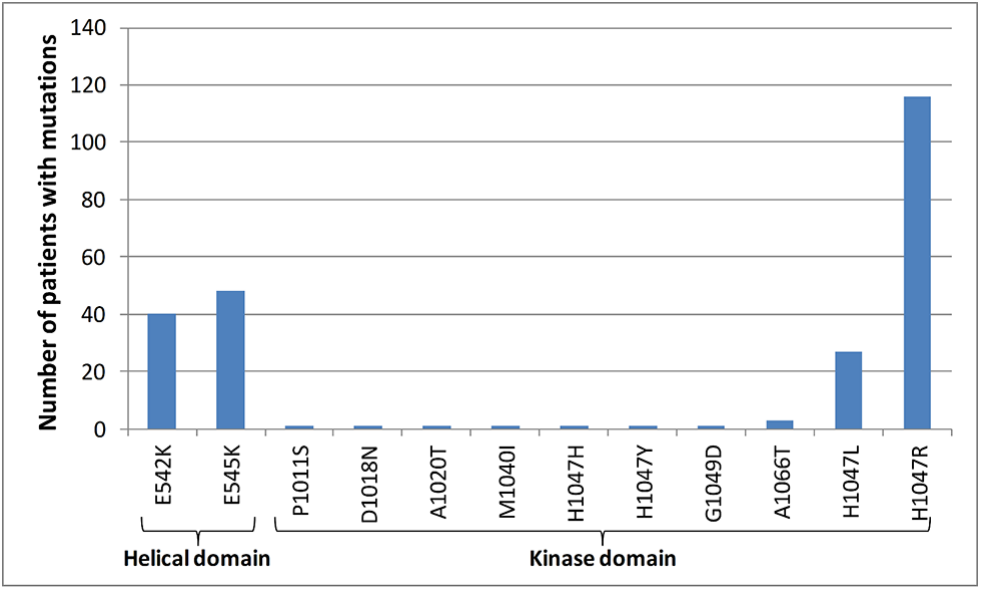
Number of patients with different types of PIK3CA mutations assessed by Sanger/qPCR in the entire population (N = 1008).

In total, tumor tissues from 1008 patients were evaluated for PIK3CA mutation detection by Sanger/qPCR (see CONSORT diagram in **Fig 1**). PIK3CA mutations were found with this classic approach in 235 (23.3%) patients. Among them, 147 (14.6%) presented with mutations in PIK3CAkin and 85 (8.4%) in PIK3CAhel, while 3 patients (0.3%) had mutations in both the helical and kinase domains (**Table 1, Fig 2**). In 610 patients (60.5%), PIK3CA mutations were assessed by both Sanger/qPCR and NGS. PIK3CA mutations with NGS were found in 149 patients (24.4%). Comparisons of mutation types and PIK3CA mutation status by both methods are presented in **S2 and S3 Tables,** respectively. Overall, the discordance rate of PIK3CA status with the two methods was 8.8%, whereas Cohen’s Kappa indicated good agreement between Sanger/qPCR and NGS results, with a coefficient of 0.76 (95% CI 0.69–0.82). In general, NGS was more sensitive than Sanger/qPCR in detecting PIK3CA mutations. In the 7 Sanger/qPCR-mutated NGS-wild-type cases the corresponding variants had been filtered out for inadequate quality metrics. In the 44 Sanger/qPCR wild type, NGS positive cases, all but one discrepant PIK3CA variants concerned the most commonly affected c.1624, c.1633, and c.3140 nucleotides with adequate read quality but with 6–12% variant allele frequency in 38/44 cases that might have been missed by the other two methods in samples with low DNA quality. Patients were classified in 3 groups according to their PIK3CA status, i.e. PIK3CA wild-type (PIK3CAwt), PIK3CA kinase domain mutant (PIK3CAkin) and PIK3CA helical domain mutant (PIK3CAhel). Patients with mutations found in both the helical and kinase domains were randomly allocated in the PIK3CAhel group for the purposes of the current analysis.

**Table 1:** PIK3CA mutation frequencies assessed by Sanger/qPCR in the entire group.

		N	%
**PIK3CA mutation status by Sanger/qPCR**			
PIK3CAwt		773	76.7
PIK3CAkin		147	14.6
PIK3CAhel		88	8.7
**Total**		1008	100.0
**PIK3CA aa change**	**PIK3CA nt change**		
H1047R	3140A>G	112	11.1
E545K	1633G>A	47	4.7
E542K	1624G>A	38	3.8
H1047L	3140A>T	27	2.7
H1047R,E542K	3140A>G,1624G>A	2	0.2
H1047R,E545K	3140A>G,1633G>A	1	0.1
H1047Y	3139C>T	1	0.1
H1047H	3142T>C	1	0.1
G1049D	3146G>A	1	0.1
P1011S	3031C>T	1	0.1
D1018N	3052G>A	1	0.1
A1066V	3197C>T hom	1	0.1
A1020T, H1047R, A1066T	3058G>A, 3140A>G, 3196G	1	0.1
M1040I, p.A1066V	3120G>A, 3197C>T	1	0.1

PIK3CAhel, mutations present in the helical (and kinase) domain; PIK3CAkin, mutations present only in the kinase domain; PIK3CAwt, PIK3CA wild-type; aa, amino acid; nt, nucleotide.

### Association of PIK3CA mutations with basic characteristics and treatment

Basic patients’ characteristics can be found in **Table 2**. All patients except 5 (0.5%) had infiltrated regional lymph node(s) at surgery and approximately equal numbers were pre- and postmenopausal. Differences in the distribution of tumor characteristics were found between the three PIK3CA groups by Sanger/qPCR (**Table 2**). PIK3CAhel tumors were more frequently smaller in size, well-moderately differentiated and of lobular histology. PIK3CAkin tumors were less frequently HER2-enriched. PIK3CAmut were significantly more prevalent in luminal tumors (ER/PgR positive, 27.1%) than in MAC (AR positive and ER/PgR negative, 16%) or hormone receptor-negative (AR/ER/PgR negative, 13%) tumors (p = 0.004). Regarding adjuvant treatment, PIK3CAkin patients were more often treated with taxane-based chemotherapy than the other two groups. This may have been caused by slight differences in the inclusion criteria of the two HeCOG trials.

**Table 2:** Patient, tumor and treatment characteristics according to PIK3CA status and the type of PIK3CA mutations, as determined by Sanger/qPCR.

		PIK3CA mutation status by Sanger/qPCR			
		PIK3CAhel	PIK3CAkin	PIK3CAwt	p-value
Patients	N	88	147	773	
Age (years)	Mean (range)	54.5 (34–76)	54.1 (28–77)	52.7 (22–79)	
		**N (%)**	**N (%)**	**N (%)**	
	<50 years	34 (39)	53 (36)	316 (41)	0.53
	≥50 years	54 (61)	94 (64)	457 (59)	
Menopausal status	Pre	45 (51)	64 (44)	356 (46)	0.53
	Post	43 (49)	83 (56)	417 (54)	
Histological grade	I-II	57 (65)	82 (56)	357 (46)	0.001
	III-Undifferentiated	31 (35)	65 (44)	416 (54)	
Histological subtype	Ductal	64 (73)	115 (78)	603 (78)	0.030
	Lobular	17 (19)	18 (12)	72 (9)	
	Mixed	5 (6)	12 (8)	57 (7)	
	Other	2 (2)	2 (2)	41 (6)	
Tumor size	≤2cm	40 (45)	40 (27)	229 (30)	0.006
	>2cm	48 (55)	107 (73)	544 (70)	
Positive lymph nodes	0	0 (0)	0 (0)	5 (1)	0.43
	1–3	35 (40)	65 (44)	289 (37)	
	≥4	53 (60)	82 (56)	479 (62)	
Subtype classification	Luminal A	29 (36)	45 (33)	150 (21)	<0.001
(N = 928)	Luminal B	29 (36)	65 (47)	275 (39)	
	Luminal-HER2	10 (12)	11 (8)	102 (14)	
	HER2-enriched	6 (7)	5 (4)	86 (12)	
	Triple-negative	7 (9)	11 (8)	97 (14)	
Breast cancer	Luminal	66 (88)	122 (89)	506 (78)	0.025
subgroups (N = 857)	MAC	6 (8)	9 (7)	79 (12)	
	HR-negative	3 (4)	6 (4)	60 (8)	
Surgery	MRM	57 (65)	101 (69)	546 (71)	0.50
	BCS	31 (35)	46 (31)	227 (29)	
Randomization group	E-CMF	22 (25)	11 (7)	140 (18)	0.003
	ET-CMF	29 (33)	64 (44)	259 (34)	
	E-T-CMF	37 (42)	72 (49)	374 (48)	
Paclitaxel treatment	Yes	66 (75)	136 (93)	633 (82)	0.001
	No	22 (25)	11 (7)	140 (18)	
Adjuvant	Yes	74 (85)	119 (86)	603 (79)	0.13
hormonotherapy	No	13 (15)	20 (14)	157 (21)	
(N = 986)					
Adjuvant radiotherapy	Yes	67 (77)	103 (74)	588 (78)	0.57
(N = 978)	No	20 (23)	36 (26)	164 (22)	

BCS, breast conserving surgery; CMF, cyclophosphamide-methotrexate-5-fluorouracil; E, epirubicin; HR, hormone receptor; MAC, molecular apocrine; MRM, modified radical mastectomy; NR, not reported; PIK3CAhel, mutations present in the helical (and kinase) domain; PIK3CAkin, mutations present only in the kinase domain; PIK3CAwt, PIK3CA wild-type; T, paclitaxel.

Similarly, PIK3CAmut as revealed with NGS were significantly more often in luminal tumors (27.8%) as compared to MAC (17.8%) and to hormone receptor negative (13.9%) tumors (p = 0.053). Helical PIK3CA mutations with this method were again associated with more favorable disease characteristics, such as histological grade I-II and Luminal A subtype (**S4 Table**).

The distribution of other biomarkers in the three PIK3CA groups is shown in **S5 Table**. Notably, EGFR positivity by IHC was observed in a decreasing order from the PIK3CAwt group to the PIK3CAkin and finally the PIK3CAhel group. Also, the frequency of IGF1R-beta high was differentially distributed between the PIK3CA groups.

The above analysis (**Table 2, S4 and S5 Tables**) was performed also excluding the 3 cases with mutations at both helical and kinase domains (data not shown). The figures were found very similar, indicating that the decision to include or not these cases in the analysis did not influence the results.

### PIK3CA mutations as prognostic factors

**Fig 3:**
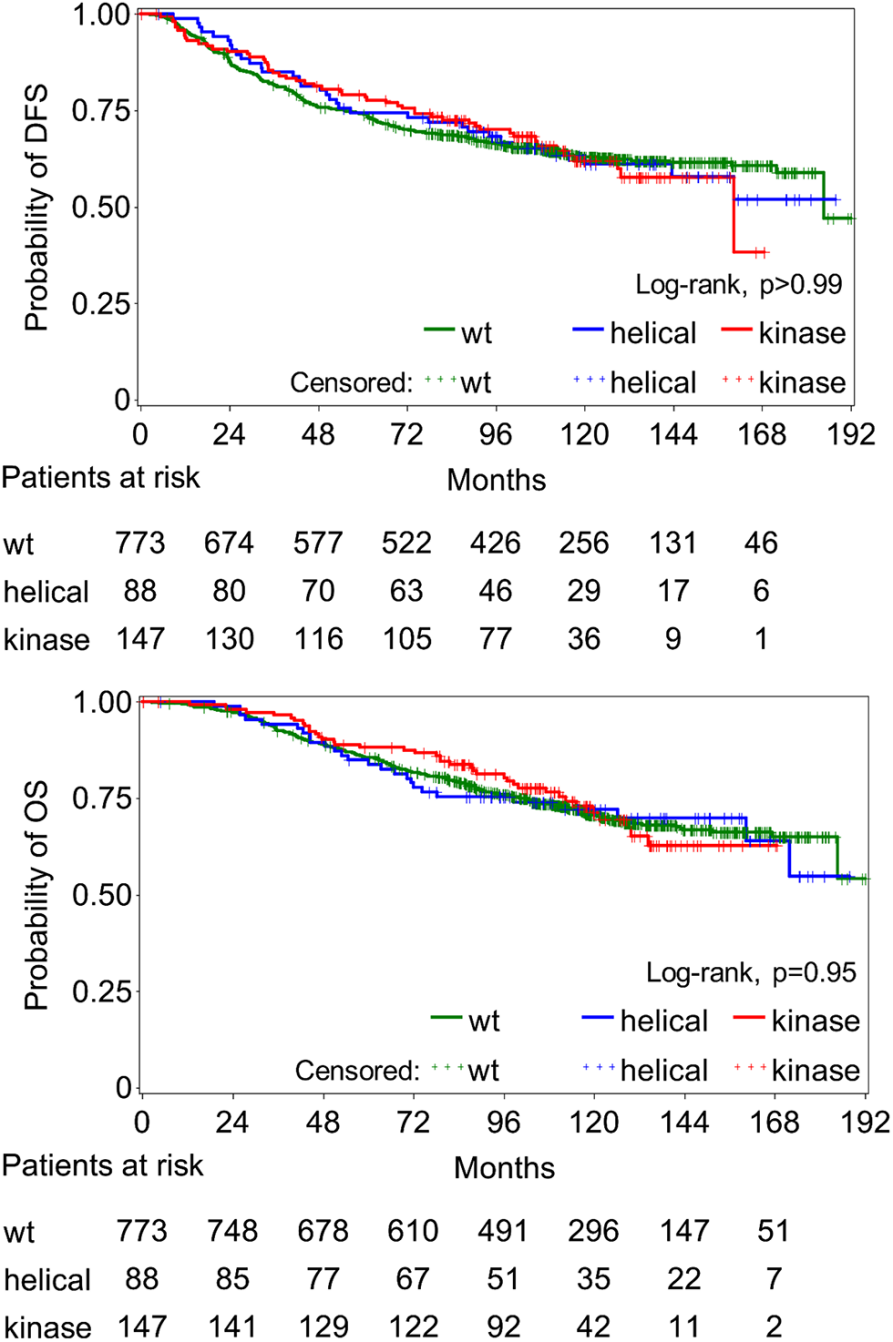
Disease-free survival (DFS) and overall survival (OS) in the entire population, according to PIK3CA status (mutations in the helical [and kinase] or kinase only domain or wild-type [wt]). No significant differences observed between the groups.

After a median follow-up of 121.9 months (range 0.1–191.9), 289 deaths (28.7%) and 365 relapses (36.2%) were recorded. Median DFS and OS did not differ significantly between PIK3CAwt and mutant tumors, while they were also similar in the 3 different PIK3CA groups, as determined with Sanger/qPCR **(Table 3)** and NGS **(S6 Table).** Survival curves of the entire population (Sanger/qPCR data) are shown in **Fig 3**.

**Table 3:** Disease-free survival (DFS) and overall survival (OS) according to the PIK3CA status (Sanger/qPCR).

	N	4-year DFS (%)	Log-rank p-value	4-year OS (%)	Log-rank p-value
All patients	1008	77.1	.	89.3	.
PIK3CAmut	235	81.3	0.99	90.0	0.89
PIK3CAwt	773	75.8		89.1	
PIK3CAhel	88	81.4	>0.99	89.5	0.95
PIK3CAkin	147	81.3		90.3	
PIK3CAwt	773	75.8		89.1	

PIK3CAhel, mutations present in the helical (and kinase) domain; PIK3CAkin, mutations present only in the kinase domain; PIK3CAwt, PIK3CA wild-type; DFS, disease-free survival; OS, overall survival.

Only Sanger/qPCR mutations were included in prognostic subgroup analyses because, as shown in **S3 Table**, categories with helical PIK3CA mutations in non-luminal subtypes were too small for reliable statistical comparisons. Univariate analysis of the prognostic significance of the PIK3CA mutation status stratified for each level of biomarker expression and breast cancer subtypes is depicted in **S7 Table**.

**Fig 4:**
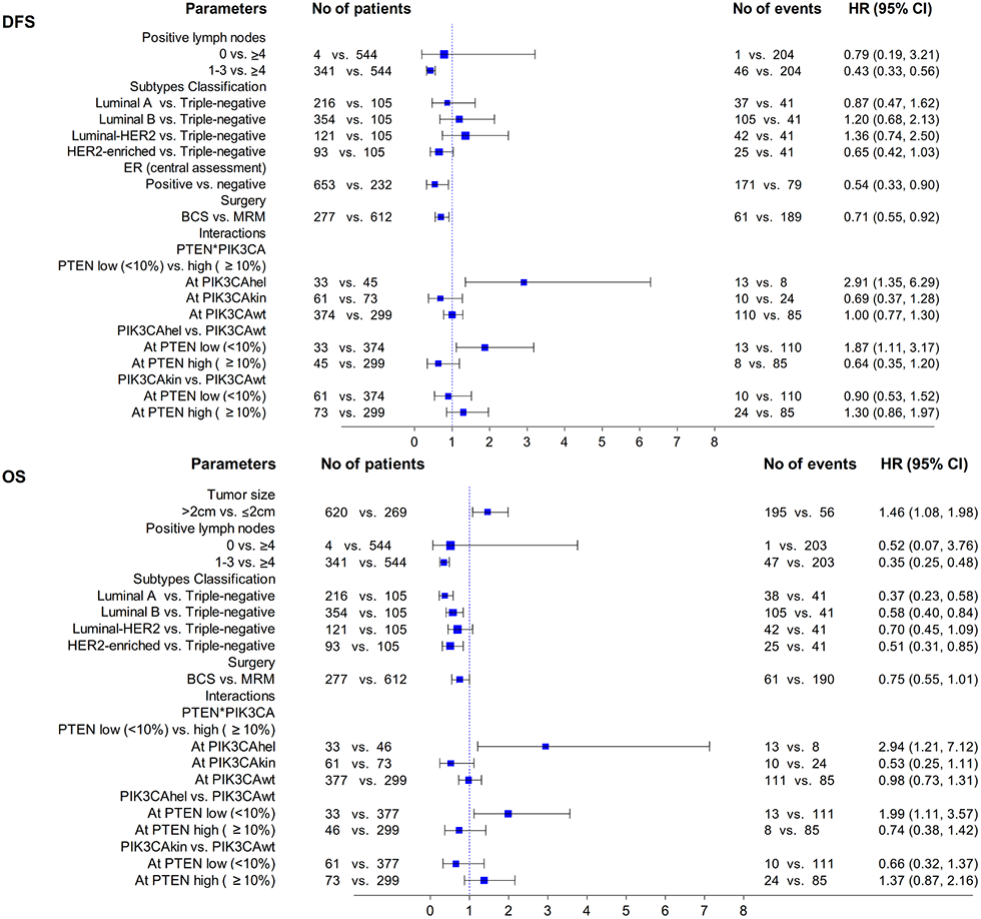
Multivariate Cox regression analysis presented by forest plots.

Multivariate analysis of prognostic factors for DFS and OS is described in **Fig 4**. Nodal stage, tumor subtype, ER status and the type of surgery were found to be important predictors of DFS, while tumor size, nodal stage and tumor subtype were prognostic for OS. Of note, a statistically significant interaction was observed between PIK3CA mutation status, as determined by Sanger/qPCR, and PTEN protein expression levels. Specifically, PIK3CAhel mutations were an adverse prognostic factor only within the group of patients with PTEN low protein expression and, inversely, PTEN low protein expression was a poor prognostic factor only in the PIK3CAhel group.

## Discussion

PIK3CA mutations are one of the most frequent genetic alterations in breast carcinomas **[18]**. Although their role has been the subject of extensive research, our knowledge is limited regarding their significance in malignant diseases. The present study has several strengths: 1) the population was retrieved from two randomized trials with very similar chemotherapy regimens including E and CMF with or without T; 2) more than half of eligible patients (1008 [62%] of the 1681 total eligible patients enrolled in the two randomized HeCOG trials described in Methods) were included in the study; 3) all except 5 patients (1%) were lymph node positive; and 4) PIK3CA mutation analysis was performed by two methods, Sanger/qPCR and NGS. Notably, Jiang et al **[19]** described intratumoral heterogeneity regarding PIK3CA mutations in 28.4% of cases. NGS is much more reliable in distinguishing tumor from normal DNA or different tumor clones **[20]**. However, the disagreement rate between the two methods was low. On the contrary, some caveats could be noticed: 1) NGS was performed in a subgroup of 610 patients, which represents 60.5% of the study population; 2) concordance between Sanger/qPCR and NGS was high but not perfect; 3) HER2 positive patients did not receive the currently standard regimen, which includes trastuzumab, although its activity did not seem to be influenced by PIK3CA genotype, as shown recently by Pogue-Geile et al **[21]**; and finally 4) the sample size was large but not big enough to offer adequate statistical power to examine small but important subgroups of patients, such as MAC or hormone receptor negative patients with different types of PIK3CA mutations.

The present study is one of the largest among those examining the role of PIK3CA mutations in breast cancer. Our PIK3CA mutation rate (23.3%) was consistent with the literature, as well as our rate of different hotspot mutations **[3, 14, 19, 22–34]**. Also, we confirmed significant associations of different types of PIK3CA mutations with clinicopathological and molecular characteristics.

One important question is the possible effect of PIK3CA status on cancer outcome and treatment efficacy. Notably, many studies examining the prognostic significance of PIK3CA mutations in breast cancer **[35]** have been published, however very few included a large enough number of patients **[3, 14, 19, 22–34]**. Also, less than half of them **[22, 24, 26, 28, 30, 31, 33]** used data from clinical trials, thus excluding the effect of treatment heterogeneity when evaluating prognostic factors. Up to now, results are largely inconclusive with some studies showing favorable and other adverse impact of PIK3CA mutations on patient outcome, while a significant number of reports did not show any prognostic significance. Moreover, recent studies incorporating patient data from big randomized studies did not show any significant effect of PIK3CA status on recurrence or survival rate **[22, 24, 26, 28]**, which has also been shown in the present study.

Preclinical models **[27, 36]** have demonstrated that PIK3CA mutations alone, compared to PTEN loss or AKT1 mutations, can cause weaker or more inconsistent activation of PI3K-AKT signaling. However, PTEN knockdown was shown to reinforce the ability of PIK3CA mutations to trigger this pathway. Also, PTEN loss increases tumor cell susceptibility to PI3K inhibition compared to PIK3CA mutations **[27]**. Therefore, PIK3CA mutations do not seem to have prognostic or therapeutic significance per se, but only in the context of other PI3K pathway aberrations.

Notably, PTEN loss and PIK3CA mutations are regarded in the literature to be the most frequent downstream molecular aberrations affecting PI3K-AKT signaling, nevertheless only a minority of studies has examined the possible role of such combined effect **[26, 31, 33, 34]**. Interestingly, each one of these studies provided different results, which may be attributed to the diverse population characteristics and treatments applied, for example only HER2 positive **[33]**, only postmenopausal **[26, 31]**, or only hormone receptor positive cases **[34]**. Also, the differences in the methodologies and/or cutoffs used in assessing PTEN protein expression may have further influenced the results as well.

Although initially there was an impression that PIK3CA mutations are mutually exclusive with PTEN loss **[37]**, this was refuted by subsequent studies **[31]**, giving a percentage of approximately 14% of coexistence of both molecular alterations, whereas in our population the coexistence of PIK3CA mutations and low PTEN protein expression was detected in 94 patients (9%). Notably, both studies **[26, 34]** that tested the prognostic significance of PTEN expression separately from PIK3CA mutations did not find any significant results, while those combining them **[31, 33]** reported their independent prognostic significance. In the present study, a significant interaction was detected between PTEN expression and PIK3CA mutational status, confirmed by multivariate analysis. This may imply that patients with tumors bearing both low PTEN protein expression and PIK3CAhel mutations may be characterized by significantly worse prognosis. However, in subgroup analysis, patients with both low PTEN expression and PIK3CAhel mutations had only marginally lower 4-year DFS and OS compared to the other subgroups (p = 0.078). Therefore, this finding is suggestive of a trend and cannot support definitive conclusions, considering also the small size (only 33 patients) of this subgroup.

## Conclusions

The present study showed that the different types of PIK3CA mutations do not seem to have any prognostic role in breast cancer patients treated with adjuvant chemotherapy. However, combining the effect of PIK3CA mutation status with that of PTEN expression levels led to the detection of statistically significant interactions with regard to prognosis. As the sample size was relatively small for subgroup analyses, we cannot exclude the possibility that these results might have occurred by chance. Nonetheless, there is strong preclinical rationale to support them and, therefore, this study offers interesting hypotheses to test in the laboratory or in larger cohorts of patients with the above characteristics.

## Supporting Information

S1 Table Clinical trial characteristics. (DOCX)

S2 Table Comparison of specific PIK3CA mutations detected by Sanger/qPCR and NGS in the subgroup of 610 patients. (DOCX)

S3 Table Comparison of PIK3CA mutation status assessed by Sanger/qPCR and NGS in the group of 610 patients. (DOCX)

S4 Table Patient, tumor and treatment characteristics according to the status of PIK3CA and the type of mutation, as determined by NGS. (DOCX)

S5 Table Biomarker expression according to the status of PIK3CA and the type of mutation assessed by Sanger/qPCR in the entire group (see REMARK diagram). (DOCX)

S6 Table Disease-free (DFS) and overall survival (OS) according to PIK3CA status assessed by NGS. (DOCX)

S7 Table Univariate analysis of the prognostic significance of PIK3CA status assessed by Sanger/qPCR stratified by biomarker. (DOCX)
