# Supplementary material for: Significance of PIK3CA Mutations in Patients with Early Breast Cancer Treated with Adjuvant Chemotherapy: A Hellenic Cooperative Oncology Group (HeCOG) Study
Source: PLoS One. 2015 Oct 9;10(10):e0140293. doi: 10.1371/journal.pone.0140293 (PMC4599795; doi:10.1371/journal.pone.0140293)
Supplement: S1 Table — (DOCX) [file pone.0140293.s001.docx]

**S1 Table: Clinical trial characteristics.**

| **Trial** | **Accrual period** | **N** | **n** | **Treatment schedule** | **Eligibility criteria** |
| --- | --- | --- | --- | --- | --- |
| HE10/97 [Fountzilas 2005] | 1997 - 2000 | 595 | 309 | **E-T-CMF**: Epirubicin 110 mg/m^2^ q 2 weeks x 3 followed by paclitaxel 250 mg/m^2^ q 2 weeks x 3 followed by cyclophosphamide 840 mg/m^2^, methotrexate 57 mg/m^2^, fluorouracil 840 mg/m^2^ (CMF) q 2 weeks x 3. GCSF support in all cycles. *vs.* **E-CMF**: Epirubicin 110 mg/m^2^ q 2 weeks x 4 followed by cyclophosphamide 840 mg/m^2^, methotrexate 57 mg/m^2^, fluorouracil 840 mg/m^2^ (CMF) q 2 weeks x 4. GCSF support in all cycles. | Eligible were women with: histologically confirmed epithelial breast cancer; pathological stage T13N1M0 or T3N0M0; Eastern Cooperative Oncology Group performance status 0-1; normal cardiac function; and adequate bone marrow, hepatic and renal function. |
| ACTRN-12611000506998 |  |  |  | Patients with ER/PgR-positive tumors received tamoxifen 20 mg daily for five years. Premenopausal patients received additional treatment with an LH-RH analog for two years. All patients who underwent partial mastectomy or with tumors >5 cm and/or with ≥4 infiltrated axillary nodes, irrespectively of the type of surgery, were irradiated. Radiation therapy and hormonal therapy were administered after the completion of chemotherapy. |  |
| HE10/00  [Gogas 2012] | 2000 - 2005 | 1,086 | 782 | **E-T-CMF**: As in the HE10/97 trial. *vs.* **ET-CMF**: Epirubicin 83 mg/m^2^ + paclitaxel 187 mg/m^2^ q 3 weeks x 4 followed by cyclophosphamide 840 mg/m^2^; methotrexate 57 mg/m^2^; fluorouracil 840 mg/m^2^ (CMF) q 2 weeks x 3. GCSF support in all cycles. | Eligible women were women with: histologically confirmed epithelial breast cancer; pathological stage T14N12M0; Eastern Cooperative Oncology Group performance status of 0-1; normal cardiac function and adequate bone marrow, hepatic and renal function. |
| ACTRN-12609001036202 |  |  |  | Premenopausal patients received hormonal therapy as in the HE10/97 trial. Postmenopausal patients received tamoxifen 20 mg daily for 2-3 years followed 2-3 years of daily examestane 25 mg. Criteria for irradiation were the same as in the HE10/97 trial. |  |

N, number of patients enrolled in the trials; n, number of patients included in the current study.
