# Supplementary material for: Significance of PIK3CA Mutations in Patients with Early Breast Cancer Treated with Adjuvant Chemotherapy: A Hellenic Cooperative Oncology Group (HeCOG) Study
Source: PLoS One. 2015 Oct 9;10(10):e0140293. doi: 10.1371/journal.pone.0140293 (PMC4599795; doi:10.1371/journal.pone.0140293)
Supplement: S2 Table — (DOCX) [file pone.0140293.s002.docx]

**S2 Table. Comparison of specific PIK3CA mutations detected by Sanger/qPCR and NGS in the subgroup of 610 patients.**

| **PIK3CA nucleotide changes**  **by Sanger/qPCR** | **PIK3CA nucleotide changes**  **by NGS** | **Frequency** | **%** |
| --- | --- | --- | --- |
| wt | wt | 454 | 74.43 |
| wt | 3140A>G | 7 | 1.15 |
| wt | 1633G>A | 11 | 1.80 |
| wt | 1624G>A | 7 | 1.15 |
| wt | 3140A>T | 7 | 1.15 |
| wt | 3118A>C | 2 | 0.33 |
| wt | 3016C>T,1633G>A | 1 | 0.16 |
| wt | 3104C>T | 1 | 0.16 |
| wt | 3133G>A,3072G>A | 1 | 0.16 |
| wt | 3140A>G,1633G>A | 1 | 0.16 |
| wt | 3148G>A | 1 | 0.16 |
| wt | 3155C>T | 1 | 0.16 |
| wt | 3159_3159delA | 1 | 0.16 |
| wt | 3170G>A,3155C>T,3153G>A | 1 | 0.16 |
| wt | 3196G>A,1633G>A | 1 | 0.16 |
| wt | 3201_3202insA | 1 | 0.16 |
| 3140A>G | wt | 4 | 0.66 |
| 3140A>G | 3140A>G | 44 | 7.21 |
| 3140A>G | 3140A>T | 2 | 0.33 |
| 3140A>G | 3019G>A | 1 | 0.16 |
| 3140A>G | 3021C>T | 1 | 0.16 |
| 3140A>G | 3140A>G,1635G>C | 1 | 0.16 |
| 3140A>G | 3140A>G,3143A>G | 1 | 0.16 |
| 3140A>G | 3170G>A,3140A>G | 1 | 0.16 |
| 1633G>A | wt | 1 | 0.16 |
| 1633G>A | 1633G>A | 22 | 3.61 |
| 1633G>A | 3012G>A | 1 | 0.16 |
| 1633G>A | 3196G>A | 1 | 0.16 |
| 1624G>A | wt | 1 | 0.16 |
| 1624G>A | 1624G>A | 17 | 2.79 |
| 1624G>A | 3136G>A,3148G>A | 1 | 0.16 |
| 3140A>T | 3140A>G | 1 | 0.16 |
| 3140A>T | 3140A>T | 10 | 1.64 |
| 3058G>A, 3140A>G , 3196G | 3140A>G | 1 | 0.16 |
| 3146G>A | wt | 1 | 0.16 |

NGS, next generation sequencing; wt, wild-type.
