# Supplementary material for: Significance of PIK3CA Mutations in Patients with Early Breast Cancer Treated with Adjuvant Chemotherapy: A Hellenic Cooperative Oncology Group (HeCOG) Study
Source: PLoS One. 2015 Oct 9;10(10):e0140293. doi: 10.1371/journal.pone.0140293 (PMC4599795; doi:10.1371/journal.pone.0140293)
Supplement: S3 Table — (DOCX) [file pone.0140293.s003.docx]

**S3 Table. Comparison of PIK3CA mutation status assessed by Sanger/qPCR and NGS in the group of 610 patients.**

|  | **PIK3CA mutations detected by NGS** | | | |
| --- | --- | --- | --- | --- |
| **PIK3CA mutations detected by Sanger/qPCR** | **PIK3CAwt**  **N (%)** | **PIK3CAhel**  **N (%)** | **PIK3CAkin**  **N (%)** | **Total** |
| **PIK3CAwt** | 454 (91) | 20 (4) | 24 (5) | 498 |
| **PIK3CAhel** | 2 (4) | 39 (89) | 3 (7) | 44 |
| **PIK3CAkin** | 5 (7) | 1 (2) | 62 (91) | 68 |
| **Total** | 461 | 60 | 89 | 610 |

NGS, next generation sequencing; PIK3CAhel, mutation(s) present in helical (and kinase) domain; PIK3CAkin, mutation(s) present only in kinase domain; PIK3CAwt, wild-type.
