# Supplementary material for: Significance of PIK3CA Mutations in Patients with Early Breast Cancer Treated with Adjuvant Chemotherapy: A Hellenic Cooperative Oncology Group (HeCOG) Study
Source: PLoS One. 2015 Oct 9;10(10):e0140293. doi: 10.1371/journal.pone.0140293 (PMC4599795; doi:10.1371/journal.pone.0140293)
Supplement: S4 Table — (DOCX) [file pone.0140293.s004.docx]

**S4 Table. Patient, tumor and treatment characteristics according to the status of PIK3CA and the type of mutation, as determined by NGS.**

|  |  | **PIK3CA mutation status by NGS** | | | |
| --- | --- | --- | --- | --- | --- |
|  |  | **PIK3CAhel** | **PIK3CAkin** | **PIK3CAwt** | **p-value** |
|  |  |  |  |  |  |
| Patients | N | 60 | 89 | 461 |  |
|  |  |  |  |  |  |
| Age (years) | Mean (range) | 53.0(36-76) | 51.4(22-77) | 53.1(24-80) |  |
|  |  | **N (%)** | **N (%)** | **N (%)** |  |
|  | <50 years | 26 (43.3) | 40 (44.9) | 182 (39.5) | 0.57 |
|  | ≥50 years | 34 (56.7) | 49 (55.1) | 279 (60.5) |  |
|  |  |  |  |  |  |
| Menopausal status | Pre | 34 (56.7) | 43 (48.3) | 208 (45.1) | 0.23 |
|  | Post | 26 (43.3) | 46 (51.7) | 253 (54.9) |  |
|  |  |  |  |  |  |
| Histological grade | I-II | 37 (61.7) | 51 (57.3) | 212 (46.0) | 0.019 |
|  | III-Undifferentiated | 23 (38.3) | 38 (42.7) | 249 (54.0) |  |
|  |  |  |  |  |  |
| Histological subtype | Ductal | 44 (73.3) | 62 (69.7) | 365 (79.2) | 0.021 |
|  | Lobular | 8 (13.3) | 16 (18.0) | 35 (7.6) |  |
|  | Mixed | 6 (10.0) | 9 (10.1) | 31 (6.7) |  |
|  | Other | 2 (3.3) | 2 (2.2) | 30 (6.5) |  |
|  |  |  |  |  |  |
| Tumor size | ≤2cm | 23 (38.3) | 24 (27.0) | 131 (28.4) | 0.25 |
|  | >2cm | 37 (61.7) | 65 (73.0) | 330 (71.6) |  |
|  |  |  |  |  |  |
| Positive lymph nodes | 0 |  | 1 (1.1) | 1 (0.2) | 0.64 |
|  | 1-3 | 21 (35.0) | 34 (38.2) | 181 (39.3) |  |
|  | ≥4 | 39 (65.0) | 54 (60.7) | 279 (60.5) |  |
|  |  |  |  |  |  |
| Subtype classification | Luminal A | 23 (39.0) | 23 (26.7) | 87 (19.7) | 0.004 |
| (N=586) | Luminal B | 20 (33.9) | 45 (52.3) | 184 (41.7) |  |
|  | Luminal-HER2 | 7 (11.9) | 8 (9.3) | 73 (16.6) |  |
|  | HER2-enriched | 5 (8.5) | 2 (2.3) | 53 (12.0) |  |
|  | Triple-negative | 4 (6.8) | 8 (9.3) | 44 (10.0) |  |
|  |  |  |  |  |  |
| Breast cancer subgroups | Luminal | 49 (89.1) | 77 (88.5) | 324 (79.8) | 0.17 |
| (N=548) | MAC | 5 (9.1) | 6 (6.9) | 51 (12.6) |  |
|  | HR negative | 1 (1.8) | 4 (4.6) | 31 (7.6) |  |
|  |  |  |  |  |  |
| Surgery | MRM | 37 (61.7) | 60 (67.4) | 319 (69.2) | 0.49 |
|  | BCS | 23 (38.3) | 29 (32.6) | 142 (30.8) |  |
|  |  |  |  |  |  |
| Randomization group | E-CMF | 10 (16.7) | 13 (14.6) | 59 (12.8) | 0.88 |
|  | ET-CMF | 21 (35.0) | 36 (40.4) | 185 (40.1) |  |
|  | E-T-CMF | 29 (48.3) | 40 (44.9) | 217 (47.1) |  |
|  |  |  |  |  |  |
| Paclitaxel treatment | Yes | 50 (83.3) | 76 (85.4) | 402 (87.2) | 0.67 |
|  | No | 10 (16.7) | 13 (14.6) | 59 (12.8) |  |
|  |  |  |  |  |  |
| Adjuvant hormonotherapy | Yes | 50 (86.2) | 74 (86.0) | 355 (78.0) | 0.11 |
| (N=599) | No | 8 (13.8) | 12 (14.0) | 100 (22.0) |  |
|  |  |  |  |  |  |
| Adjuvant radiotherapy | Yes | 51 (87.9) | 68 (80.0) | 352 (78.7) | 0.26 |
| (N=590) | No | 7 (12.1) | 17 (20.0) | 95 (21.3) |  |

BCS, breast conserving surgery; CMF, cyclophosphamide-methotrexate-5-fluorouracil; E, epirubicin; HR, hormone receptor; MAC, molecular apocrine; MRM, modified radical mastectomy; NR, not reported; PIK3CAhel, mutation(s) present in helical (and kinase) domain; PIK3CAkin, mutation(s) present only in kinase domain; PIK3CAwt, wild-type; T, paclitaxel.
