# Supplementary material for: Significance of PIK3CA Mutations in Patients with Early Breast Cancer Treated with Adjuvant Chemotherapy: A Hellenic Cooperative Oncology Group (HeCOG) Study
Source: PLoS One. 2015 Oct 9;10(10):e0140293. doi: 10.1371/journal.pone.0140293 (PMC4599795; doi:10.1371/journal.pone.0140293)
Supplement: S5 Table — (DOCX) [file pone.0140293.s005.docx]

**S5 Table. Biomarker expression according to the status of PIK3CA and the type of mutation assessed by Sanger/qPCR in the entire group (see REMARK diagram).**

|  | | **PIK3CA mutation status** | | | |
| --- | --- | --- | --- | --- | --- |
|  | | **PIK3CAhel**  **N (%)** | **PIK3CAkin**  **N (%)** | **PIK3CAwt**  **N (%)** | **p-value** |
|  | | | | | |
| EGFR | Negative (<1%) | 80 (95) | 125 (90) | 581 (81) | 0.001 |
|  | Positive (≥1%) | 4 (5) | 14 (10) | 133 (19) |  |
|  |  |  |  |  |  |
| IGF1R-alpha | High | 28 (35) | 42 (32) | 200 (30) | 0.61 |
|  | Low | 52 (65) | 90 (68) | 469 (70) |  |
|  | | | | | |
| IGF1R-beta | High | 15 (25) | 22 (23) | 192 (37) | 0.017 |
|  | Low | 44 (75) | 72 (77) | 333 (63) |  |
|  | | | | | |
| IGF2R | High | 34 (47) | 46 (41) | 262 (45) | 0.69 |
|  | Low | 38 (53) | 65 (59) | 315 (55) |  |
|  | | | | | |
| IGFBP2 | High | 38 (49) | 75 (55) | 324 (49) | 0.41 |
|  | Low | 39 (51) | 61 (45) | 339 (51) |  |
|  | | | | | |
| pAKT473 (cytoplasmic) | Negative | 35 (46) | 58 (44) | 287 (42) | 0.81 |
|  | Positive | 41 (54) | 75 (56) | 391 (58) |  |
|  | | | | | |
| pAKT473 (nuclear+cytoplasmic) | Either positive | 43 (57) | 83 (62) | 447 (66) | 0.23 |
|  | Both negative | 33 (43) | 50 (38) | 231 (34) |  |
|  | | | | | |
| pAKT473 (nuclear) | Negative | 58 (76) | 103 (77) | 525 (77) | 0.98 |
|  | Positive | 18 (24) | 30 (23) | 153 (23) |  |
|  | | | | | |
| pAKT308 | Negative | 15 (20) | 16 (12) | 120 (18) | 0.21 |
|  | Positive (int 2-3) | 60 (80) | 117 (88) | 554 (82) |  |
|  |  |  |  |  |  |
| PTEN | Low (<10%) | 33 (42) | 61 (45) | 377 (56) | 0.011 |
|  | High (≥10%) | 46 (58) | 73 (55) | 299 (44) |  |
|  | | | | | |
| mTOR | Negative (<1%) | 14 (18) | 25 (19) | 193 (28) | 0.020 |
|  | Positive (≥1%) | 64 (82) | 108 (81) | 493 (72) |  |
|  | | | | | |
| AR | Negative (<1%) | 9 (13) | 14 (12) | 114 (21) | 0.055 |
|  | Positive (≥1%) | 58 (87) | 100 (88) | 436 (79) |  |

PIK3CAhel, mutations present in the helical (and kinase) domain; PIK3CAkin, mutations present only in the kinase domain; PIK3CAwt, PIK3CA wild-type; IGF1R, insulin-like growth factor receptor 1; IGF2R, insulin-like growth factor receptor 2; IGFBP2, insulin-like growth factor-binding protein 2; AR, androgen receptor; HR, hormone receptor; luminal, luminal A or B or HER2; MAC, molecular apocrine, HR negative, ER, PgR and AR negative.
