# Supplementary material for: Significance of PIK3CA Mutations in Patients with Early Breast Cancer Treated with Adjuvant Chemotherapy: A Hellenic Cooperative Oncology Group (HeCOG) Study
Source: PLoS One. 2015 Oct 9;10(10):e0140293. doi: 10.1371/journal.pone.0140293 (PMC4599795; doi:10.1371/journal.pone.0140293)
Supplement: S6 Table — (DOCX) [file pone.0140293.s006.docx]

**S6 Table.** **Disease-free (DFS) and overall survival (OS) according to PIK3CA status assessed by NGS.**

|  | **N** |  | **4-year DFS (%)** | **Log-rank**  **p-value** |  | **4-year OS (%)** | **Log-rank p-value** |
| --- | --- | --- | --- | --- | --- | --- | --- |
| All patients | 610 |  | 78.5 | . |  | 89.9 | . |
|  |  |  |  |  |  |  |  |
| PIK3CAmut | 149 |  | 79.7 | 0.47 |  | 87.8 | 0.94 |
| PIK3CAwt | 461 |  | 78.1 |  |  | 90.6 |  |
|  |  |  |  |  |  |  |  |
| PIK3CAhel | 60 |  | 81.4 | 0.44 |  | 89.8 | 0.59 |
| PIK3CAkin | 89 |  | 78.7 |  |  | 86.5 |  |
| PIK3CAwt | 461 |  | 78.1 |  |  | 90.6 |  |

PIK3CAhel, mutations present in the helical (and kinase) domain; PIK3CAkin, mutations present only in the kinase domain; PIK3CAwt, PIK3CA wild-type; DFS, disease-free survival; OS, overall survival.
