# Supplementary material for: Significance of PIK3CA Mutations in Patients with Early Breast Cancer Treated with Adjuvant Chemotherapy: A Hellenic Cooperative Oncology Group (HeCOG) Study
Source: PLoS One. 2015 Oct 9;10(10):e0140293. doi: 10.1371/journal.pone.0140293 (PMC4599795; doi:10.1371/journal.pone.0140293)
Supplement: S7 Table — (DOCX) [file pone.0140293.s007.docx]

**S7 Table.** **Univariate analysis of the prognostic significance of PIK3CA status assessed by Sanger/qPCR stratified by biomarker.**

| **Strata** | **PIK3CA status** | **N** | **4-year DFS (%)** | **Log-rank**  **p-value** | **4-year OS (%)** | **Log-rank**  **p-value** |
| --- | --- | --- | --- | --- | --- | --- |
| Total sample | PIK3CAwt | 773 | 75.8 | 1.00 | 89.1 | 0.95 |
|  | PIK3CAhel | 88 | 81.4 |  | 89.5 |  |
|  | PIK3CAkin | 147 | 81.3 |  | 90.3 |  |
| Luminal A | PIK3CAwt | 150 | 88.6 | 0.24 | 94.6 | 0.23 |
|  | PIK3CAhel | 29 | 89.7 |  | 93.1 |  |
|  | PIK3CAkin | 45 | 79.1 |  | 90.7 |  |
| Luminal B | PIK3CAwt | 275 | 79.4 | 0.97 | 92.7 | 0.99 |
|  | PIK3CAhel | 29 | 85.7 |  | 92.9 |  |
|  | PIK3CAkin | 65 | 82.8 |  | 89.1 |  |
| Luminal-HER2 | PIK3CAwt | 102 | 70.5 | 0.19 | 91.2 | 0.41 |
|  | PIK3CAhel | 10 | 80.0 |  | 90.0 |  |
|  | PIK3CAkin | 11 | 63.6 |  | 90.9 |  |
| HER2-enriched | PIK3CAwt | 86 | 68.3 | 0.22 | 89.4 | 0.14 |
|  | PIK3CAhel | 6 | 50.0 |  | 66.7 |  |
|  | PIK3CAkin | 5 | 100.0 |  | 100.0 |  |
| Triple-negative | PIK3CAwt | 97 | 57.7 | 0.13 | 71.1 | 0.16 |
|  | PIK3CAhel | 7 | 33.3 |  | 66.7 |  |
|  | PIK3CAkin | 11 | 81.8 |  | 90.9 |  |
| AR negative (<1) | PIK3CAwt | 114 | 64.0 | 0.41 | 78.1 | 0.29 |
|  | PIK3CAhel | 9 | 62.5 |  | 87.5 |  |
|  | PIK3CAkin | 14 | 84.6 |  | 92.3 |  |
| AR positive (≥1) | PIK3CAwt | 436 | 80.3 | 0.55 | 92.6 | 0.56 |
|  | PIK3CAhel | 58 | 80.7 |  | 87.7 |  |
|  | PIK3CAkin | 100 | 78.8 |  | 89.9 |  |
| Luminal (A, B, HER2) | PIK3CAwt | 506 | 82.1 | 0.34 | 94.2 | 0.48 |
|  | PIK3CAhel | 66 | 86.2 |  | 92.3 |  |
|  | PIK3CAkin | 122 | 79.8 |  | 89.9 |  |
| MAC (molecular apocrine) | PIK3CAwt | 79 | 69.2 | 0.16 | 89.7 | 0.12 |
|  | PIK3CAhel | 6 | 40.0 |  | 60.0 |  |
|  | PIK3CAkin | 9 | 88.9 |  | 88.9 |  |
| HR negative (ER, PgR and AR negative) | PIK3CAwt | 60 | 56.7 | 0.29 | 70.0 | 0.12 |
|  | PIK3CAhel | 3 | 33.3 |  | 66.7 |  |
|  | PIK3CAkin | 6 | 83.3 |  | 100.0 |  |
| EGFR-negative (<1) | PIK3CAwt | 581 | 78.7 | 0.92 | 92.2 | 0.85 |
|  | PIK3CAhel | 80 | 82.1 |  | 89.7 |  |
|  | PIK3CAkin | 125 | 80.3 |  | 89.3 |  |
| EGFR-positive (≥1) | PIK3CAwt | 133 | 63.6 | 0.48 | 78.0 | 0.18 |
|  | PIK3CAhel | 4 | 50.0 |  | 75.0 |  |
|  | PIK3CAkin | 14 | 85.7 |  | 100.0 |  |
| IGF1R-alpha high and EGFR negative | PIK3CAwt | 166 | 81.9 | 0.65 | 94.0 | 0.96 |
|  | PIK3CAhel | 25 | 92.0 |  | 100.0 |  |
|  | PIK3CAkin | 38 | 86.5 |  | 91.9 |  |
| IGF1R-alpha and EGFR else | PIK3CAwt | 496 | 76.3 | 0.61 | 89.0 | 0.98 |
|  | PIK3CAhel | 53 | 77.6 |  | 84.3 |  |
|  | PIK3CAkin | 92 | 81.7 |  | 90.0 |  |
| IGF1R-alpha high and IGF2R low | PIK3CAwt | 86 | 79.1 | 0.065 | 88.4 | 0.11 |
|  | PIK3CAhel | 10 | 90.0 |  | 100.0 |  |
|  | PIK3CAkin | 14 | 100.0 |  | 100.0 |  |
| IGF1R-alpha and IGF2R else | PIK3CAwt | 471 | 76.3 | 0.61 | 90.6 | 0.67 |
|  | PIK3CAhel | 59 | 77.6 |  | 87.9 |  |
|  | PIK3CAkin | 95 | 81.7 |  | 89.2 |  |
| PTEN low (<10) | PIK3CAwt | 377 | 77.3 | 0.10 | 89.6 | 0.077 |
|  | PIK3CAhel | 33 | 65.6 |  | 84.4 |  |
|  | PIK3CAkin | 61 | 85.0 |  | 93.3 |  |
| PTEN high (≥10) | PIK3CAwt | 299 | 74.4 | 0.15 | 89.9 | 0.18 |
|  | PIK3CAhel | 46 | 91.1 |  | 93.3 |  |
|  | PIK3CAkin | 73 | 78.9 |  | 88.7 |  |

PIK3CAhel, mutations present in the helical (and kinase) domain; PIK3CAkin, mutations present only in the kinase domain; PIK3CAwt, PIK3CA wild-type; AR, androgen receptor; HR, hormone receptor; IGF1R, insulin-like growth factor receptor 1; IGF2R, insulin-like growth factor receptor 2.
